# Community Safety Needs and Resources and Their Alignment: A Case Study

**DOI:** 10.1007/s11524-026-01069-z

**Published:** 2026-05-11

**Authors:** Rose Kagawa, Alex Kwong, Colette Smirniotis, Karina Patwardhan, Emily Chan, Jeremy Prim, Paul Gruenewald, Garen Wintemute

**Affiliations:** 1 Centers for Violence Prevention, Department of Emergency Medicine/VPRP, University of California, Davis, 4150 V Street, Suite 2100, Sacramento, CA 95817 USA; 2 Department of Sociology, Social Sciences and Humanities, University of California, Davis, 1283, Davis, CA 95616 USA; 3 Prevention Research Center, 2030 Addison Street, Suite 410, Berkeley, CA 94704 USA

**Keywords:** Community safety ecosystem, Crime prevention, Firearm violence

## Abstract

Our study objective was to describe the match between community safety needs and resources in Cleveland, Ohio from 2015–2019. We conducted an extensive review of online sources, agency websites, and implementation documents to identify violence prevention and community safety activities. We coded each census tract based on the presence or absence of each specific activity type and explored cross-sectional associations with crime frequency and type, including violent, property, and firearm crime, using Besag-York-Mollie (BYM) spatial autoregressive models. We identified 1,147 activities taking place in 1,625 locations. Efforts to improve the physical environment and after-school and out-of-school programs were the most common activity types. The total number of activities per population was positively associated with crime frequency (Relative Risk (RR) = 1.14; 95% Credible Interval (CI) = 1.102, 1.176), the property crime rate (RR = 1.17; 95% CI = 1.123, 1.226), the violent crime rate (RR = 1.62; 95% CI = 1.449, 1.806), and the rate of firearm-involved violent crime (RR = 4.79; 95% CI = 2.965, 7.745). There is a strong foundation for coordinated violence prevention in Cleveland, Ohio, targeted on average, though imperfectly, to the areas with more violence.

**Supplementary Information:**

The online version contains supplementary material available at 10.1007/s11524-026-01069-z.

## Introduction

Violent injury with a firearm is the leading cause of death and disability for children and young adults 10–29 years of age [1], and effective approaches to preventing violence are urgently needed. Midsized cities, with populations ranging from 250,000 to one million, experienced the highest rates of violent crime and firearm violence over the past decade, often surpassing rates observed in the height of the crime wave of the 1990 s [2]. Yet these cities are often overlooked in research and media reporting [2]. Additionally, rates of violence vary across neighborhoods within cities, highlighting variation in need for intervention and prevention [3].

Firearm violence is a complex problem that requires multifaceted solutions. Prevention occurs along multiple continuums, from programs that support families in raising emotionally healthy children to those that intervene with adolescents and adults actively engaged in violence, and from those that work directly with individuals to those that seek to change the community conditions that lead to violence [4]. Additionally, an individual program’s success results in part from its connection with others. For example, community violence intervention (CVI) programs like Cure Violence connect individuals with the services and supports they need to heal and ultimately shift their life trajectory away from violence. Services and supports may include substance use treatment, career training, housing, and job opportunities. To fully meet the needs of individuals in CVI programs, these services and supports must be available in the community.

More generally, communities abundant in opportunity are safer communities [5]. Research has found lower rates of gun violence at schools in areas with higher employment rates and greater job security [6]. In economically and socially disadvantaged areas typically subject to higher rates of firearm violence, employment opportunities and redevelopment projects appear to serve as protective factors [5]. There is increasing recognition that law enforcement alone cannot solve the problem of violence and that comprehensive solutions are needed to address the many factors that give rise to violence. A safety ecosystem, constructed from multisector approaches to building opportunity, promoting peace, and supporting healing, can provide a more holistic approach to public safety [7].

Ideally, the community services and policies that make up the safety ecosystem arise in response to and are co-located with community needs. If the ecosystem is effective, these efforts will eventually resolve the community needs that led to their formation, creating more equitable access to safety, wellbeing, and opportunity. In the current study, “need” is marked by the occurrence of violence, and we explore the degree to which different categories of community services are positively related to rates of violent and firearm-involved crime, as would be the case if the safety ecosystem is strongest in the areas where there is greatest community need. Yet the location of services, particularly those without an explicit violence prevention aim, may be responsive to other, related community conditions. Historically, underinvestment and disinvestment, particularly in Black, Latinx, and Native American communities, have created unequal access to opportunity and wellbeing and fostered conditions for violence [8]. As such, we additionally explore the degree to which the locations of services and resources are related to neighborhood racial segregation, residential stability, and neighborhood economic segregation.

This study takes place in Cleveland, Ohio, an industrial powerhouse in the early twentieth century that, following economic restructuring, now faces many of the same challenges as other mid-sized cities across the United States. Cleveland’s population decreased dramatically during the second half of the twentieth century, primarily due to outmigration [9], and Cleveland is ranked in the top ten cities with the highest violent crime rates according to crime data from the FBI [10]. Cleveland is also home to the nation’s first community foundation (The Cleveland Foundation) and numerous community-building efforts, demonstrating a commitment to the city’s wellbeing.

Understanding the strength and character of the community safety ecosystem and the degree to which services are co-located with need can inform our understanding of the chances of successful violence prevention and the equity of opportunity. Practitioners familiar with similar activities and efforts in their own cities can draw comparisons to this example, noting where there may be similarities or differences that can inform their own service types, distribution, and patterns.

## Methods

### Study Population

Our study population included all neighborhoods in Cleveland, Ohio with a population greater than zero. The primary unit of analysis was census tracts (*N* = 175 based on 2010 census tracts), chosen because much of our data were measured in this geography or could cleanly be aggregated to this geography. However, census tracts do not necessarily correspond to socially meaningful units, sometimes amalgamating areas with vastly different physical, social, and economic experiences [11]. As such, in secondary analyses we used named neighborhoods (*N* = 34), which may be more recognizable to city residents. Research also points to the importance of multi-scale analyses of crime outcomes, noting that effects may operate differently at different levels of resolution [12].

### Data

#### Safety Ecosystem Elements

We sought to identify the presence and locations of all eligible violence prevention and community safety activities in Cleveland, Ohio during the study period (2015–2019). To be eligible for inclusion, the activity had to be free of cost for all individuals or groups that benefited from it or subsidized for low-income beneficiaries, recognized by experts in the field as a method employed to prevent violence or foster safety, designed or implemented such that neighborhood variation was possible (policies and programs that uniformly targeted national, state, or city populations were excluded), and fully operational at some point during the study period.

We began by identifying several activity categories using the Centers for Disease Control and Prevention Technical Package for the Prevention of Youth Violence and a report by the Research and Evaluation Center at John Jay College of Criminal Justice [13, 14]. These categories included for example, youth mentoring programs, counseling and therapeutic care, and improvements to the physical environment. We added categories to fill identified gaps (e.g. law enforcement actions) or adapted categories so they were not restricted to “youth”. Over the course of data collection, categories were split, combined, added, or removed based on the specifics of the programs, policies, and efforts identified. The complete list of 16 categories is available in Appendix 1.

We reviewed publicly available sources to identify activities that fit within these category descriptions. Searches began by entering key words based on each category definition in Google’s search engine. Common sources of information included news media, Facebook pages, annual reports, organization websites, and funder lists. Information discovered in one resource often led to new resources in a chain search. When otherwise unavailable, dates of operation were checked by reviewing earlier versions of websites recorded in Internet Archive. When activity eligibility was unclear, we discussed its inclusion as a team and refined the eligibility criteria as needed.

Individual team members were responsible for compiling an initial activity list for each category. When a second reviewer failed to find three new potential activities within 30 min of searching, we considered the category complete. Otherwise, data collection for the category continued with search recommendations from the second reviewer. Categories often underwent several rounds of “3 in 30” reviews, each time with a different reviewer, before completion. Once complete, each entry was reviewed by a different team member to make sure it fit within the category definition and eligibility criteria and to confirm all required information was complete.

We geocoded address, zip code, or neighborhood information and placed each activity within a census tract using 2010 census boundaries. Activities were summed by census tract to create a count of unique activities by category in each census tract. Counts were then divided by the target populations in each census tract (e.g. mentoring programs were divided by the population age 0–18 in a census tract) to generate rates of activities per capita.

#### Neighborhood Characteristics

The main explanatory variables included crime type and frequency. Crime types included violent crimes (murder, rape, robbery, and aggravated assaults), firearm-involved violent crimes, and property crimes (burglary, larceny-theft, motor vehicle theft, and arson) reported to police per 100 residents. While not the focus of this study, exploring relationships with property crimes allowed us to provide a more complete picture of how services relate spatially to crime. Crime incident reports were provided by the Cleveland Police Department. We combined violent and property crimes to get an overall measure of crime frequency. Crime rates were averaged across the study years (2015–2019). However, the method of reporting firearm-involvement changed in 2016. As such, firearm-involved crimes exclude those occurring in 2015.

We additionally explored associations with neighborhood racial segregation, residential stability, and neighborhood economic status. We used the Index of Concentrations at the Extremes (ICE) to capture Black–white residential segregation. This measure proved better able to capture associations between fatal and nonfatal assaults and residential segregation than the Dissimilarity Index in previous research [15]. The formula is as follows:

$$IC{E}_{race,ethnicity}=(W-B)/{T}_{re}$$

where *W* is the number of people who self-identify as white non-Hispanic, *B* is the number of people who self-identify as Black (Hispanic and non-Hispanic), and *T* is the total population with race and ethnicity data.

We used the ICE for income to measure neighborhood economic residential segregation. In this case, the number of people living in low-income households (L) was subtracted from the number of people living in high-income houses (H), and the difference was divided by the total population with income data (T). High- and low-income thresholds are typically set at the 20th and 80th US household income percentiles [15]. In 2010, these values were < $25,000 and ≥ $100,000 [16]. The formula is as follows:

$$IC{E}_{income}=(H-L)/{T}_{i}$$

We followed the example of the National Neighborhood Crime Study (NNCS) to measure residential instability [17]. The NNCS defines residential instability using the average of two standardized values: (1) the percentage of occupied housing units that are renter-occupied and (2) the percentage of the population 5 and over who lived in a different residence in the year prior.

With the exception of crime data, variables were sourced from the American Community Survey with interpolations by Geolytics Inc.

### Analysis

We used descriptive statistics and visualizations, primarily maps, to describe variation in safety ecosystems across neighborhoods. We also estimated associations between each of our neighborhood characteristics (crime type and frequency, neighborhood racial segregation, residential stability, and neighborhood economic segregation), modeled individually, and each category in the safety ecosystem (*n* = 16), modeled as counts of category activities per census tract, controlling for the area in square miles of the census tract and including the size of the target population as an offset. We also used total activities and activity variety (number of unique categories) as outcomes. In subsequent models, we additionally controlled for multiple neighborhood characteristics to explore how results differed. We assessed these associations using a Besag–York–Mollie (BYM) spatial autoregressive model, assuming binary queen’s adjacencies with an unstructured exchangeable component, and implemented in R (INLA, Integrated Nested Laplacian Approximations) [18]. In sensitivity analyses, we modeled these associations using negative binomial regression models.

Additional sensitivity analyses included census tracts we identified as outliers (> 5 standard deviations from the mean for > 1 category) and replaced census tracts with named neighborhoods as the unit of analysis.

## Results

The study sample included 175 census tracts with population size greater than zero. We identified 1147 activities taking place in 3743 unique activity-locations over the study period. Enhancing the physical environment (e.g. community gardens) was the most prevalent category with 811 activity-locations, followed by afterschool and out-of-school programs (e.g. tutoring, theater, sports) (*n* = 607) and enhancing the community environment (e.g. neighborhood development initiatives) (*n* = 591). Notably, some categories included programming over a wide area rather than in defined locations (e.g. community violence interventions) and may have lower total counts as a result. Table 1 provides counts and example activities in each category. Moran’s I statistics are available in Appendix Table 1.

**Table 1:** Category examples and counts over 2015–2019

Categories	Example activities	Total activities	Total locations^1^	Total activity-locations
Enhance physical environment	Community gardens, community clean-ups, tree planting, lights installation, murals, storefront renovations	149	480	811
After and out of school programs	Homework time, career exploration, sports activities, tutoring, chess club, music and performing arts	291	429	607
Enhance community environment	Home purchasing or rental support and incentives for living in specific neighborhoods, home maintenance support for seniors, loan forgiveness/financial assistance/technical or professional support for businesses in specific neighborhoods, cross-sector coalitions for crime prevention, additional funding for schools to improve education and social supports	53	111	591
Job readiness programs	Career exploration opportunities, internships/apprenticeships, resume preparation, ACT/SAT/GED support, skills training, computer classes, interview preparation	174	331	423
Community-justice system relations	Coffee, fishing, cookouts, Easter egg hunts, brunch, basketball, pancake breakfasts, chili cook-offs etc. shared between police officers and community members; co-responder pilot programs; regular community meetings with police	162	187	358
Mentoring programs	Big Brothers Big Sisters, college and career mentoring, sports-focused mentoring, mentoring for youth at risk of violence	37	153	277
Early childhood education	Preschool programs	55	143	213
Crisis services	Homeless services, shelters, clothing distribution, free meals, personal hygiene products, school supplies, dental and medical care	93	118	130
Community violence intervention	Hospital-based violence intervention programs; credible messengers engaged in mediating conflict, connecting to services, and mentorship	5	5	124
Counseling and therapeutic care	Outpatient counseling, school-based counseling, residential treatment, arts and music therapy, group therapy	35	80	91
Law enforcement action	Police raids and drug bust operations; elevated police presence at pools, recreation centers, Cavaliers parade	35	86	87
School-based socioemotional learning programs	Peer mediation, anti-bullying curricula, socioemotional learning through theater and art	20	4	84
Parent support and family relationship programs	Parenting education and support groups, counseling for families in conflict, family story time at the library, fatherhood programs	59	64	84
Substance use treatment	Opioid overdose education, naloxone distribution, detox services, residential treatment	36	46	50
Reduce youth access to and use of firearms	Gun melts, gun safety education for children, youth-led campaign to end gun violence	4	14	37
Community violence education and organizing	Neighbors organizing to reduce crime, identification and rectification of community service gaps in areas experiencing crime and violence, youth-led campaign to end gun violence	5	6	15
Total		1147	1625	3743

^1^Locations can be a specific place like a school or library (latitude longitude) or they can be neighborhoods if an activity is more dispersed. Each only counts once. If multiple locations within a neighborhood are present but not known, they are missing from this count

Of the activity-locations listed in Table 1, 182 (4.8%) lacked sufficient geographic specificity to be placed accurately in census tracts. These were sourced mainly from a grassroots grantmaking organization, NeighborUp Cleveland, and were excluded from the following analyses unless otherwise noted.

Two census tracts met criteria for outliers and were removed from the main regression analyses. These census tracts had the smallest population sizes (populations = 165 and 71, respectively; mean population = 2225), and their activity counts were above the 80th percentile.

In the following sections, we report on how neighborhood characteristics were associated with the density per population of safety ecosystem activities. The count of activities in each safety ecosystem category is the dependent variable while we explore crime frequency and type, and sociodemographic variables as independent variables.

### Associations between Neighborhood Crime Frequency and Type and the Density of the Safety Ecosystem

**Fig. 1:**
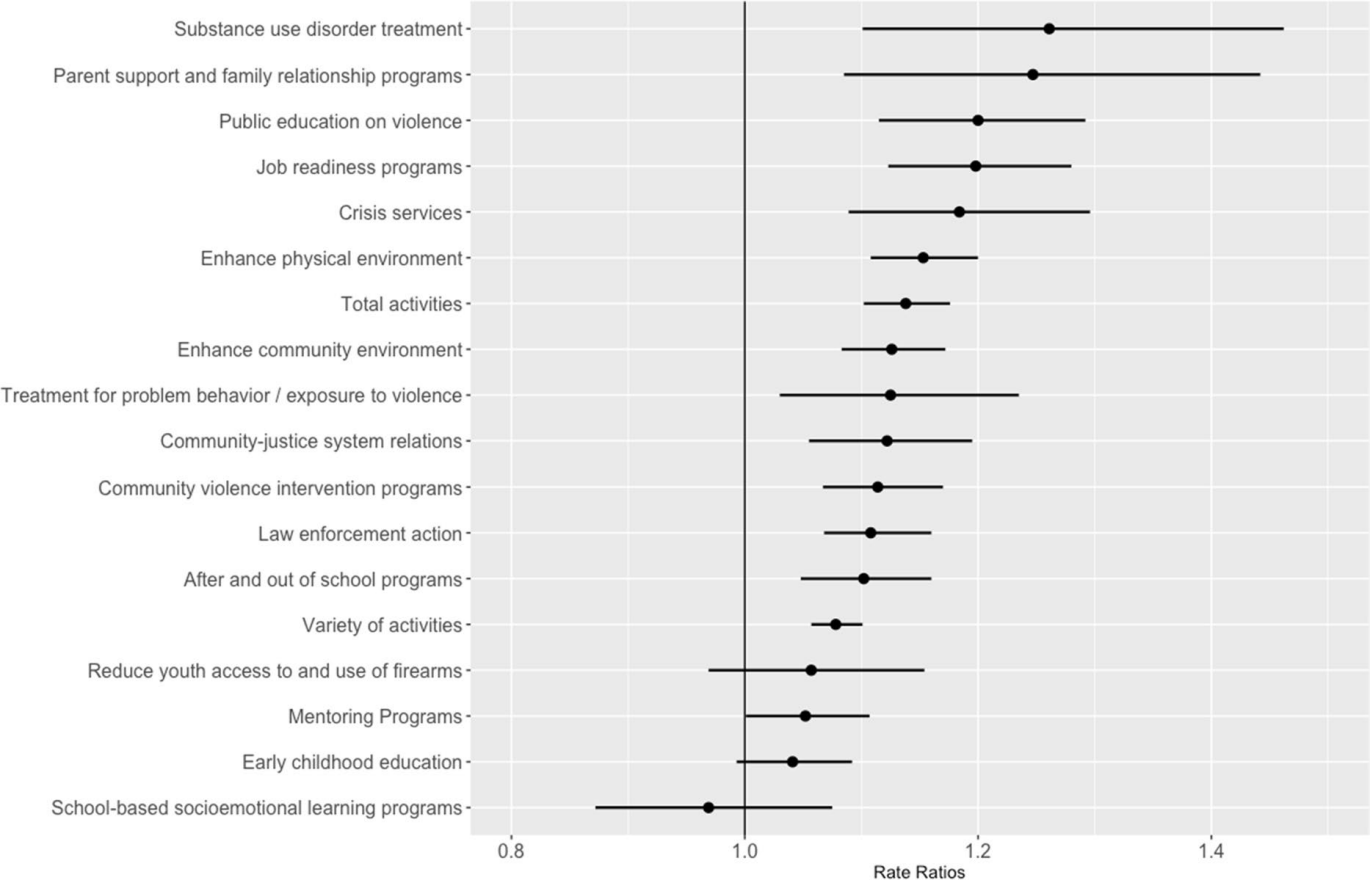
Associations between crime frequency and ecosystem categories. Rate ratios and 95% credible intervals from each of 18 models are shown. All models adjust for the area of the census tract in square miles and include a population offset, where the population is defined by the intended audience for each activity type (e.g., early childhood education uses a population offset of 0–5 year olds). Crime frequency includes violent and property crimes per resident population in the census tract. The vertical line indicates the null

Census tracts with higher rates of crime (violent and property) per population tended to have a greater density of safety ecosystem activities (Fig. 1). This was the case for the total activity count and for most individual ecosystem categories. Substance use treatment services and family relationship programs had the strongest positive associations with the crime rate, the violent crime rate, and the property crime rate (Fig. 1, Appendix Figs. 1 and 2). Universal school-based programs that support socio-emotional learning were the only program type to show a small negative, though not statistically significant, association with crime frequency and type. Associations tended to be greater in magnitude for violent crime than for property crime rates (Appendix Figs. 1 and 2).

**Fig. 2:**
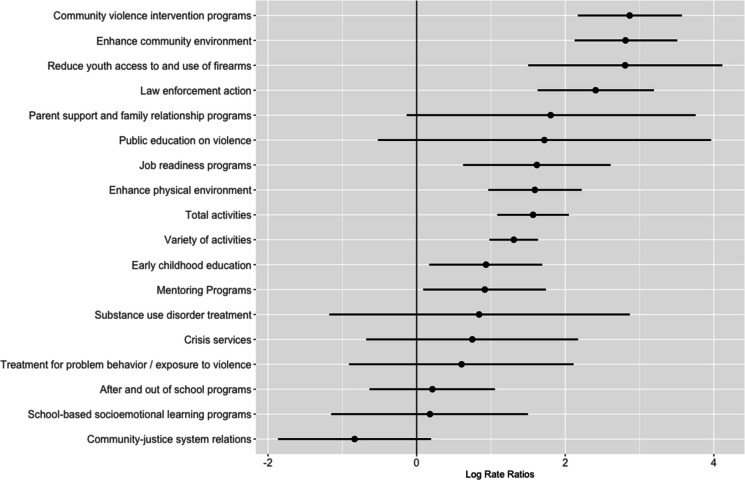
Associations between firearm violent crime rates and ecosystem categories. Data from 2015 are excluded due to changes in coding firearm involvement in 2016. Logscale rate ratios and 95% credible intervals from each model are shown for display purposes. All models adjust for the area of the census tract in square miles, and include a population offset, where the population is defined by the intended audience for each activity type (e.g., early childhood education uses a population offset of 0–5 year olds). Firearm crime rates are measured per resident population in the census tract. The vertical line at zero indicates the null

Areas with higher rates of violent, firearm-involved crimes, compared with areas with lower rates of such crimes, were also more likely to have a greater number of ecosystem activities (Fig. 2). However, the ecosystem categories that appeared most strongly associated with rates of violent, firearm-involved crime included community violence intervention programs, upstream efforts to enhance the community environment, activities related to firearm availability (e.g., gun melts), and police raids and operations. Several activity types were not geographically associated with firearm crime rates.

**Fig. 3:**
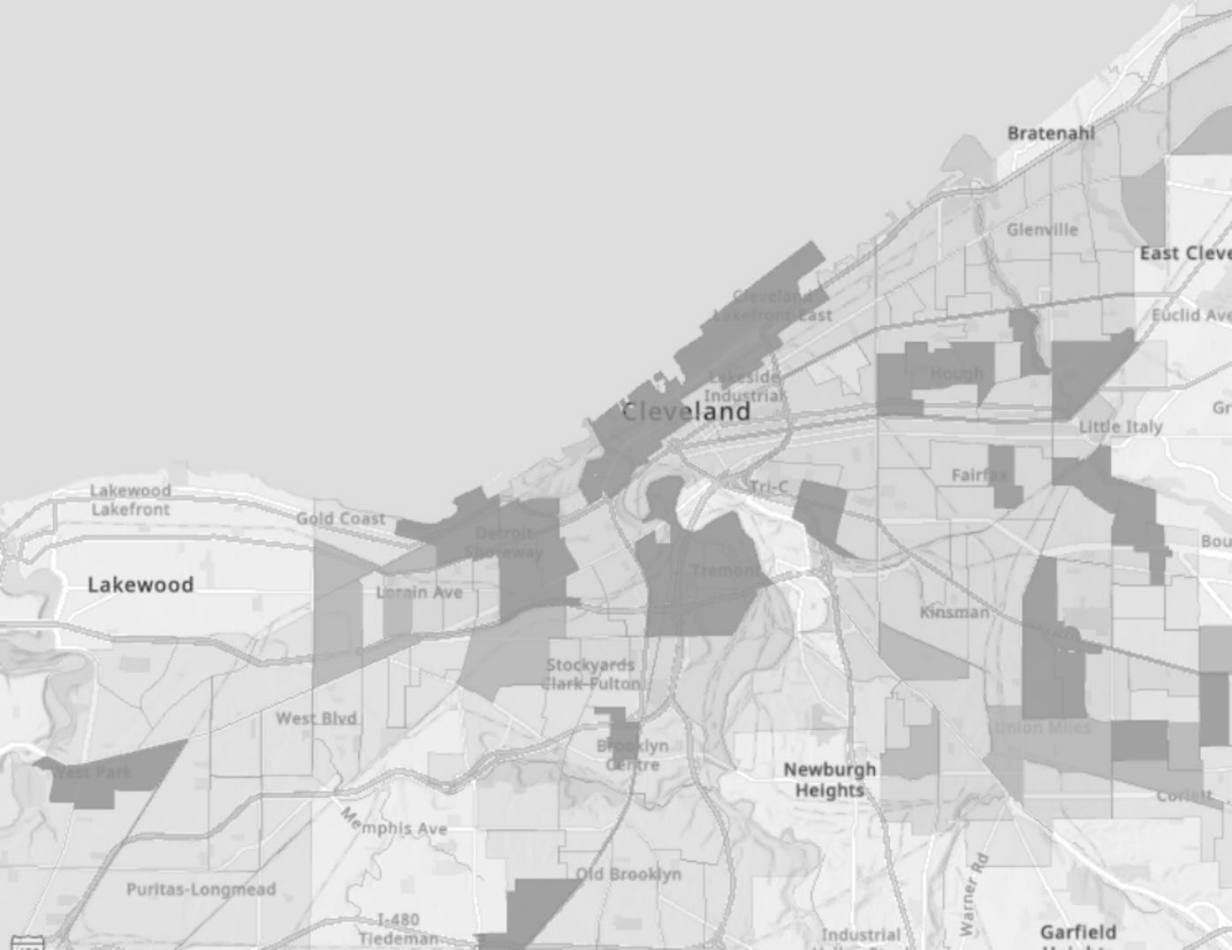
Areas of discordance between rates of violent crime and ecosystem density. Rates of violent crime > median and number of activities per population < median shown in medium gray. Rates of violent crime < median and number of activities per population > median shown in dark gray

While most ecosystem categories tended to be located in areas with elevated rates of violent and firearm-involved crime *on average*, there was discordance in the data that indicated some areas were “underserved” while others were “overserved”. Many neighborhoods had rates of violent crime that were above the median, but the number of activities per population was below the median, while other neighborhoods showed the reverse pattern (Fig. 3).

**Fig. 4:**
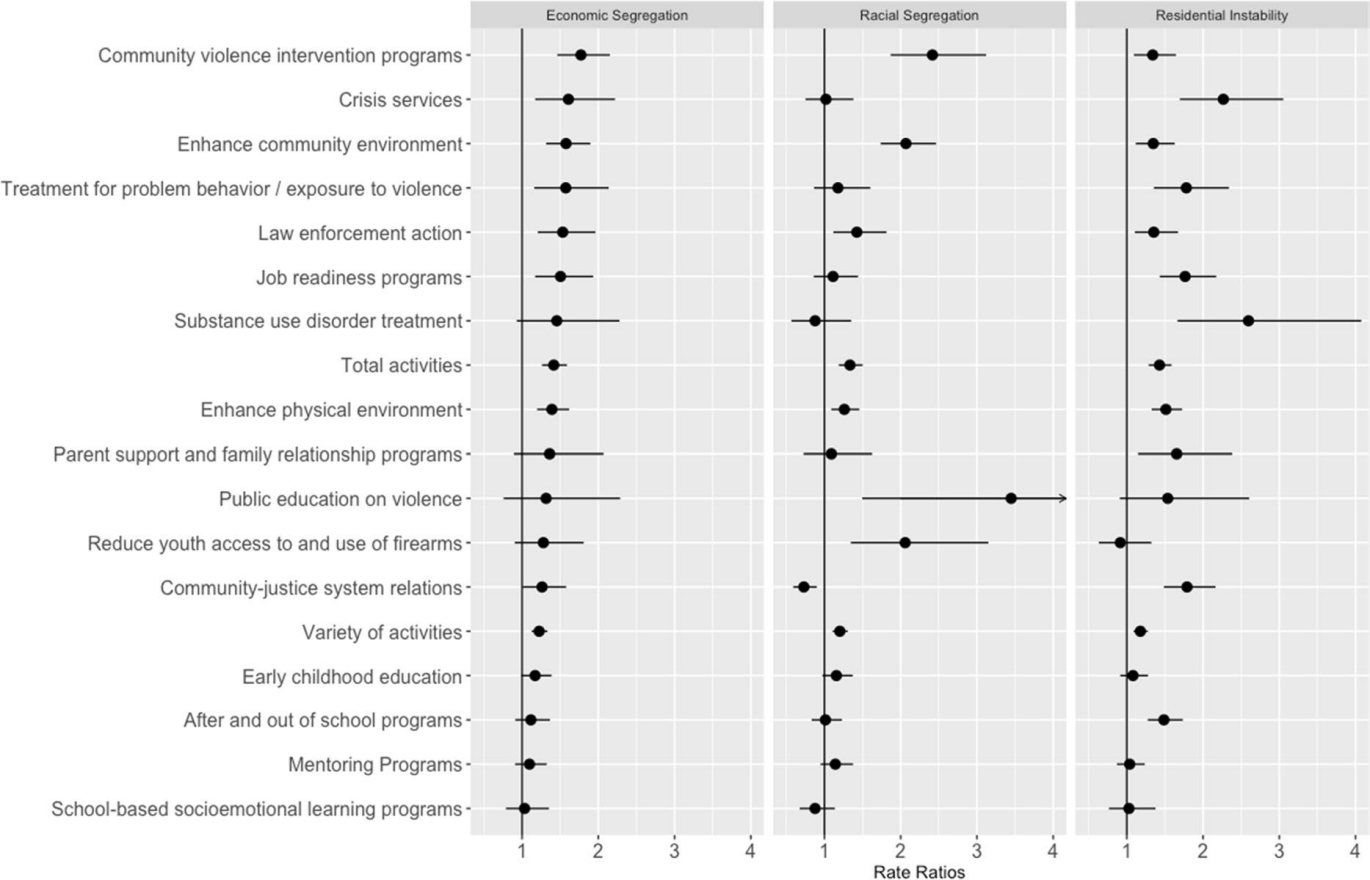
Associations between neighborhood characteristics and ecosystem categories. Rate ratios and 95% credible intervals from each model are shown. Credible intervals that extend beyond the figure are noted with an arrow. All models adjust for the area of the census tract in square miles and include a population offset, where the population is defined by the intended audience for each activity type (e.g., early childhood education uses a population offset of 0–5 year olds). Residential instability, economic segregation, and racial segregation have all been standardized with mean = 0 and standard deviation = 1 to facilitate comparisons. The vertical lines indicate the null

Rates of violent crime were higher in neighborhoods characterized by residential instability, concentrated poverty, and in those with greater Black (vs. white) segregation. Areas with greater residential instability, concentrated poverty, and Black segregation were also more likely to have a greater concentration of many safety ecosystem activities (Fig. 4). The types of activities with the greatest concentration varied across each of these measures.

#### Secondary and Sensitivity Analyses

Using the 34 named neighborhoods in Cleveland in place of census tracts as the unit of analysis, associations with crime frequency and type tended to be in a similar direction and still statistically significant, but close to the null. In the neighborhood-level models exploring associations with racial and economic segregation, the credible intervals included the null with few exceptions. Only associations with residential instability remained similar in magnitude and the degree to which they were statistically distinguished from the null. These results were similar whether we included all identified activities, or only those for which a census tract could be identified, the latter approach being more parallel to the census tract analysis.

When the two outlier census tracts were included in sensitivity analyses, results were often attenuated though similar. However, in our models for violent, firearm-involved crime, the magnitude of the associations was much smaller, and the credible intervals were far narrower, resulting in several differences in the parameter estimates. These census tracts had the two highest rates of violent, firearm-involved crime (16.6 and 95.8 per 1,000, compared with a mean of 3.5). The census tract with the highest rate in particular served as an influential point, whose inclusion had a large effect on parameter estimates (Appendix Fig. 3).

Finally, the patterns noted in our main analyses were very similar when using negative binomial regression models.

## Discussion

This study provides a comprehensive description of a safety ecosystem in a post-industrial Midwestern city with high rates of violence and firearm violence. We found a dense and geographically dispersed array of activities, programs, and policies with the potential for impacting violence. Our results show activities were more concentrated, on average, in areas with higher rates of violence; this was particularly true for firearm violence. These findings suggest services were often located in the areas where they were most needed. At the same time, the match between ecosystem activities and rates of crime was imperfect, with some neighborhoods that experience low levels of violence receiving numerous activities and some neighborhoods that experience high levels of violence receiving fewer activities.

The most prevalent categories were efforts to enhance the physical environment, after and out-of-school programs, and community-level efforts to foster safe and healthy neighborhoods. The activities included in these categories are rooted in evidence-based primary prevention efforts. The physical environments surrounding people are associated with individual probabilities of engaging in or being the victim of violence. For example, evidence suggests landscape factors such as neighborhood greenspace [19], city lighting [20], and transportation design [21] are associated with firearm violence and related factors such as aggression and crime. Research also shows that organizational norms and the policies and programs organizations implement (e.g. housing and lending practices [22], stop and frisk policies [23], policies regulating the density and type of alcohol outlets [24, 25]) directly shape the neighborhood environment in ways that can foster safety or reduce risk factors for violence. The density of efforts to lay the foundation for socially and economically healthy neighborhoods reflects a commitment to making sustainable change in violence-impacted areas of Cleveland.

While far less prevalent overall, community violence intervention programs tended to be among those most strongly associated with rates of violence and firearm violence. similar programs are growing in use across the country, with many evaluations suggesting beneficial effects for those involved [26, 27], though others have identified mixed results on violence risk [28]. Additionally, CVI programs rely on the broader service environment (e.g. job readiness programs, substance use treatment, housing and food supports, and therapeutic care for victims of violence) to meet the varied and individually defined needs of the populations they serve. While our findings suggest such services were somewhat more available in areas with more crime overall, except for job readiness programs, they were not as clearly targeted to areas with higher rates of firearm violence specifically. Nor were other direct service programs that are not traditionally targeted to those involved in violence: activities to support new and struggling families, after and out-of-school programs, and universal school-based socioemotional programs. We did not have information on reach or unmet needs in the community, but such services are often highly impacted with long waitlists, highlighting the importance of increasing availability of services in the areas of greatest need.

We also found police raids and operations were more concentrated in areas with higher rates of violence. A large body of research suggests greater police presence is associated with modest reductions in crime [29, 30]. Police raids and operations are thought to deter criminal activity by demonstrating the consequences of engaging in illegal behaviors [31]. The presence of police raids and operations in areas with elevated rates of firearm violence mirrors evidence of the effectiveness of concentrating police efforts in crime hot spots [32]. At the same time, there is growing recognition of the harms caused by aggressive policing [33, 34]. How police work within communities is critically important to police effectiveness and community wellbeing [35].

Importantly, efforts to foster trust between community members and the criminal legal system were not more concentrated in areas with elevated rates of firearm violence. To the extent that such efforts are effective at reducing community harm caused by police and improving police ability to meet a community’s safety needs, this is a missed opportunity. Such trust-building efforts were, however, geographically associated with police actions such as raids and operations.

Our main analyses used census tracts as the unit of analysis to identify associations within relatively small geographic areas. Associations were far weaker when using Cleveland neighborhoods as the unit of analysis. Cleveland neighborhoods cover much larger geographic areas than census tracts, and at this level of spatial resolution, rates of violent crime and firearm violence were less variable. We hypothesize the scale of these larger units hid important local variation. We did not explore other levels of spatial resolution, which may show different patterns of associations.

Additional limitations include our lack of data on program outputs (e.g. # workshops held, # people served), the quality of the activities identified, and other contextual information that would be important for understanding the strength of the safety ecosystem in a more nuanced manner. It is possible a census tract is home to many low-quality activities that serve a small audience, while another is home to only a few activities, but they are impactful for a wide population. Such variation is missed in the current study. Finally, while we sought to identify all relevant activities during our study period, our search strategy relied on public information available via the internet and we are likely missing eligible activities as a result.

In this case study, numerous and varied activities work to prevent and respond to violence. These activities were more often located in the areas of greatest need, as measured by reports of violent and firearm-involved crime, but alignment was imperfect, with several census tracts with elevated crime rates receiving few services. This detailed and nuanced picture of a safety ecosystem informs how violence prevention is conceptualized and put into action and highlights the broader concepts of community safety and opportunity that are necessary for human thriving.

## Supplementary Information

Below is the link to the electronic supplementary material.

ESM 1 (DOCX 268 KB)
