# Supplementary material for: Community Safety Needs and Resources and Their Alignment: A Case Study
Source: J Urban Health. 2026 May 11;103(3):512–23. doi: 10.1007/s11524-026-01069-z (PMC13315026; doi:10.1007/s11524-026-01069-z)
Supplement: Supplementary file 1 — (DOCX 268 KB) [file 11524_2026_1069_MOESM1_ESM.docx]

**Appendix 1. Approach Categories**

Enhance the physical environment. Activities that alter the physical environment with the goals of reducing visual disorder, creating environments that promote mental health and well-being, and encouraging social interaction and the forging of stronger connectedness and collective efficacy. Examples include community clean-ups, mural creation, and community gardens and parks.

Enhance the community environment. Efforts to create an environment that maximizes opportunity and well-being and minimizes community-level risks. Neighborhood initiatives to foster a positive social and physical environment include efforts to create local opportunities for stable employment, infrastructure for community gardens or farmers markets, and new/small business support. Reducing exposure to risks includes efforts to create affordable housing, the provision of a guaranteed income, or minimum wage increases, for example. This is distinguished from other activities in its focus on the “upstream” conditions (e.g. changing land use codes vs. creating a community garden).

After-school and out-of-school programs. Programs that provide youth with positive activities and social connections with peers and mentors during otherwise unstructured times after school, on weekends, and during the summer. Programs are often designed to support social and academic skill development, increase connections with school and community, and build supportive relationships with peers and adults.

Job readiness programs. Programs that prepare individuals to join the workforce through skill development and experience acquisition. Programs for high school age audiences or older were considered.

Community and justice system relations. Programs and activities designed to build connection and trust between community members and police officers, policies or policing strategies that encourage communication and listening, trainings for police officers designed to support community-oriented policing, and policies or practices that increase policing and judicial transparency.

Mentoring programs. Mentoring programs pair youth with caring adults with the goal of supporting the personal and academic growth of the young person.

Early childhood education. Early education programming (e.g. preschool or pre-Kindergarten) for children ages 0-5 from economically disadvantaged families.

Crisis services. The provision of free or subsidized support to address urgent physical (e.g. food, clothing, shelter) and mental (e.g. counseling) health needs. To be eligible, services must be regularly available in order to address crises as they occur.

Community violence intervention (CVI). CVI approaches use credible messengers to engage individuals at high risk of violence involvement (as perpetrators or victims). Credible messengers may help mediate conflicts, provide individualized mentorship, and connect youth to needed supports and services in the community. Hospital-based violence intervention programs, which use hospital visits for violence-related injuries as the initial point of contact, are also included in this category.

Counseling and therapeutic care. Counseling and therapeutic care for those who have been exposed to violence and those exhibiting problem behavior or risk factors for involvement in violence. Such care can help mitigate the mental health and behavioral consequences of witnessing violence as well as help youth build positive problem-solving and emotional management skills.

Law enforcement actions. The actions police take to enforce the law. This includes the detection of criminal activity, through surveillance or police deployment, and the apprehension of suspects through police operations and arrests.

School-based socioemotional learning programs. Universal programs offered within schools to children and adolescents with the goal of developing emotional and interpersonal skills. Topics may include problem solving and conflict management, emotional awareness and regulation, communication, and empathy.

Parenting support and family relationship programs. Services provided to parents, caregivers, or families that aim to improve family functioning and positive parenting practices. Skill building may focus on communication, child developmental stages and expectations, behavioral monitoring, and problem solving. This category also includes programs that help prepare new parents and caregivers for their role.

Substance use treatment. Counseling, behavioral therapy, or medication-assisted care provided with the goal of helping individuals reduce their dependence on substances such as alcohol, prescription drugs, or illicit drugs.

Reduce youths’ unsupervised access, possession, and use of firearms. Efforts to stymie illicit firearm markets, increase safe storage (unloaded and locked) in homes, remove firearms from high-risk homes, or reduce the availability of firearms more generally.

Community violence education and organizing. Efforts to promote community norms about the unacceptability of violence, educate the public about the effects of violence in the community, and inspire community members to view violence as preventable and take action to prevent its occurrence.

**Appendix Table 1. Spatial Autocorrelation**

| Category | Morans I | P-Value |
| --- | --- | --- |
| **Enhance physical environment** | **0.07** | **0.048** |
| **Enhance community environment** | **0.06** | **0.035** |
| After and out of school programs | -0.004 | 0.450 |
| Job readiness programs | -0.008 | 0.379 |
| **Community & justice system relations** | **0.07** | **0.059** |
| Mentoring programs | -0.008 | 0.515 |
| Early childhood education | -0.06 | 0.925 |
| Crisis services | -0.0006 | 0.364 |
| Community violence intervention | 0.007 | 0.230 |
| Counseling and therapeutic care | -0.03 | 0.797 |
| Law enforcement action | -0.02 | 0.588 |
| School-based socioemotional learning programs | -0.04 | 0.751 |
| Parent support and family relationship programs | -0.007 | 0.427 |
| Substance use treatment | -0.002 | 0.341 |
| **Reduce youth access to and use of firearms** | **0.18** | **0.001** |
| **Community violence education and organizing** | **0.16** | **0.003** |
| Total | 0.01 | 0.126 |

**Appendix Figure 1.** **Associations Between Violent Crime Rates and Ecosystem Categories**

**
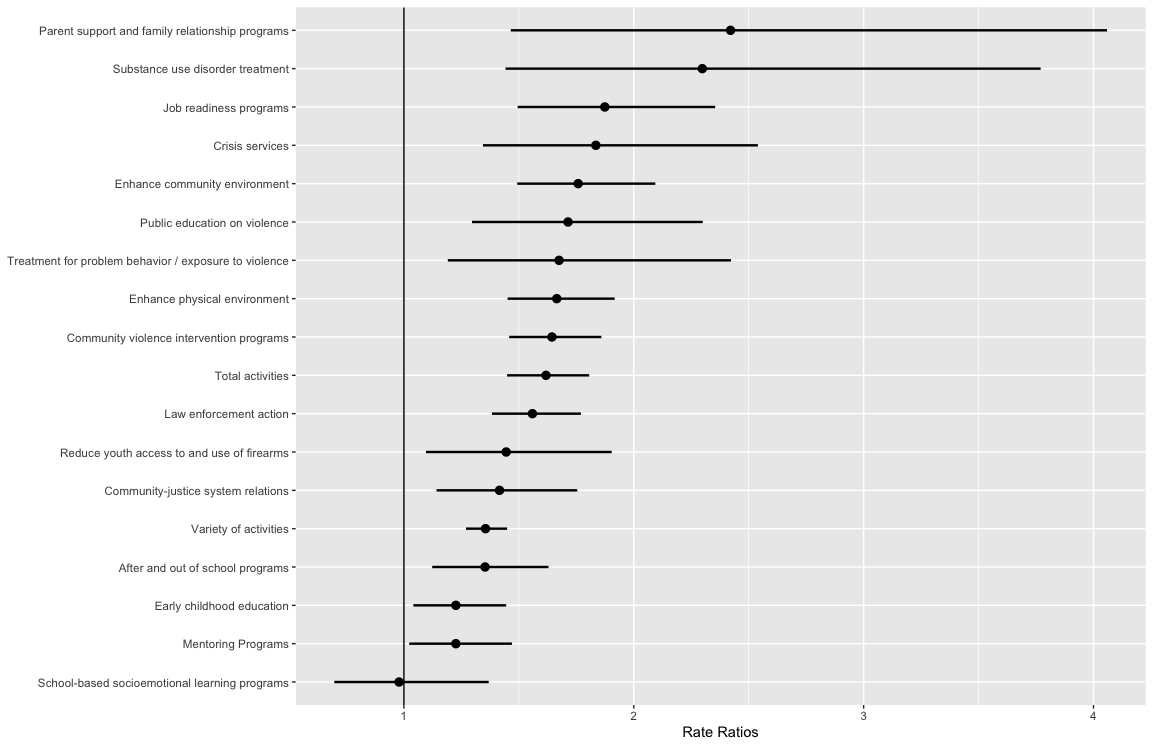
**

**
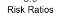
**

Note. Rate ratios and 95% credible intervals from each of 18 models are shown. All models adjust for the area of the census tract in square miles and include a population offset, where the population is defined by the intended audience for each activity type (e.g., early childhood education uses a population offset of 0-5 year olds). The vertical line indicates the null.

**Appendix Figure 2. Associations Between Property Crime Rates and Ecosystem Categories**

**
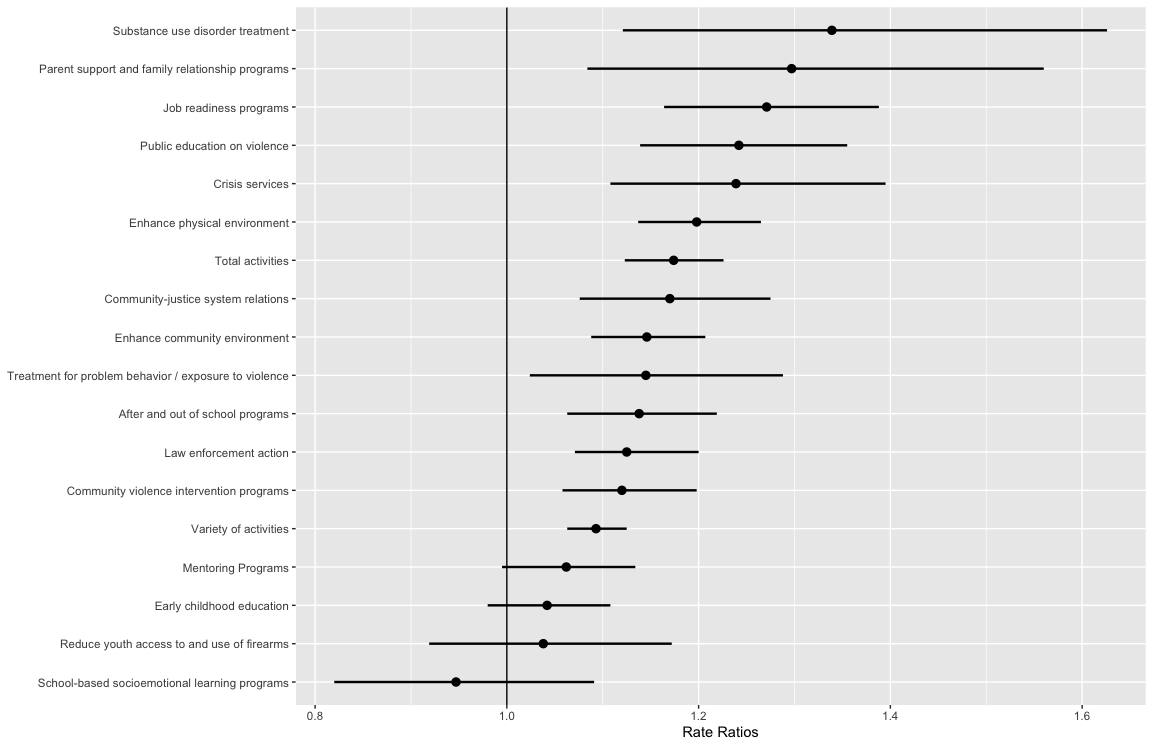
**

**
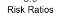
**

Note. Rate ratios and 95% credible intervals from each of 18 models are shown. All models adjust for the area of the census tract in square miles and include a population offset, where the population is defined by the intended audience for each activity type (e.g., early childhood education uses a population offset of 0-5 year olds). The vertical line indicates the null.

**Appendix Figure 3. Associations Between Firearm Violent Crime Rates and Ecosystem Categories with All 175 Census Tracts Included**


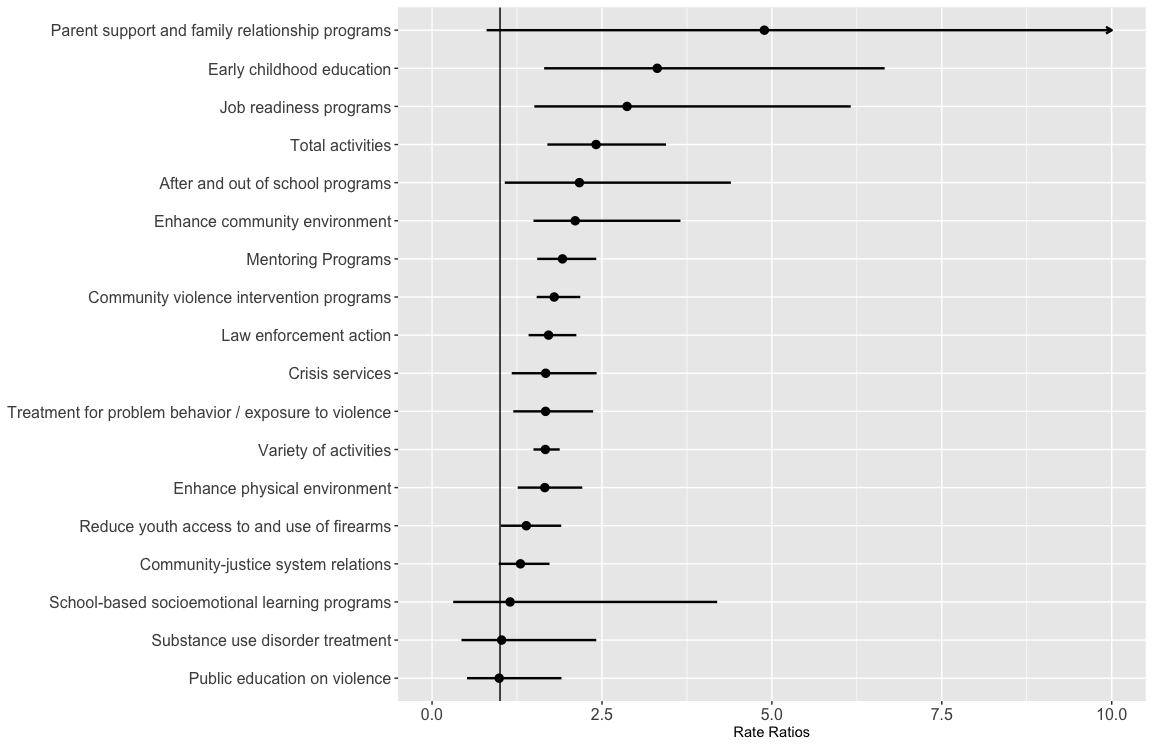


Note. Rate ratios and 95% credible intervals from each of 18 models are shown. All models adjust for the area of the census tract in square miles and include a population offset, where the population is defined by the intended audience for each activity type (e.g., early childhood education uses a population offset of 0-5 year olds). The vertical line indicates the null.
